# Quantifying the spatial nonstationary response of influencing factors on ecosystem health based on the geographical weighted regression (GWR) model: an example in Inner Mongolia, China, from 1995 to 2020

**DOI:** 10.1007/s11356-023-26915-4

**Published:** 2023-05-16

**Authors:** Li Na, Yu Shi, Luo Guo

**Affiliations:** 1 College of Life and Environmental Sciences, Minzu University of China, Beijing, 100081 China; 2 State Key Laboratory of Urban and Regional Ecology, Research Center for Eco-Environmental Sciences, Chinese Academy of Sciences, Beijing, 100085 China

**Keywords:** Ecosystem health, Influencing factor, Geographically weighted regression, Inner Mongolia

## Abstract

The identification of ecosystem health and its influencing factors is crucial to the sustainable management of ecosystems and ecosystem restoration. Although numerous studies on ecosystem health have been carried out from different perspectives, few studies have systematically investigated the spatiotemporal heterogeneity between ecosystem health and its influencing factors. Considering this gap, the spatial relationships between ecosystem health and its factors concerning climate, socioeconomic, and natural resource endowment at the county level were estimated based on a geographically weighted regression (GWR) model. The spatiotemporal distribution pattern and driving mechanism of ecosystem health were systematically analysed. The results showed the following: (1) the ecosystem health level in Inner Mongolia spatially increases from northwest to southeast, displaying notable global spatial autocorrelation and local spatial aggregation. (2) The factors influencing ecosystem health exhibit significant spatial heterogeneity. Annual average precipitation (AMP) and biodiversity (BI) are positively correlated with ecosystem health, and annual average temperature (AMT) and land use intensity (LUI) are estimated to be negatively correlated with ecosystem health. (3) Annual average precipitation (AMP) significantly improves ecosystem health, whereas annual average temperature (AMT) significantly worsens eco-health in the eastern and northern regions. LUI negatively impacts ecosystem health in western counties (such as Alxa, Ordos, and Baynnur). This study contributes to extending our understanding of ecosystem health depending on spatial scale and can inform decision-makers about how to control various influencing factors to improve the local ecology under local conditions. Finally, this study also proposes some relevant policy suggestions and provides effective ecosystem preservation and management support in Inner Mongolia.

## Introduction

Urbanization in China has made remarkable progress during the past 40 years, and the urbanization rate has more than doubled by 57.35% in 2017 compared to 17.92% in 1978. The average urbanization rate in China from 1978 to 2017 was approximately 2.45 times greater than that of the world during the same period (Ren et al. 2018). From the beginning of the twenty-first century to 2015, the urbanization rate in Inner Mongolia rose by 60.3%. High-speed urbanization has led to significant landscape pattern alterations, which have altered ecosystem structure and function (Huilei et al. 2017). As the world’s second-largest economy, China has made remarkable achievements in its economic development. Following the exploitation of the western region, Inner Mongolia has become one of China’s fastest developing provinces and the second largest coal-producing province (Xiao et al. 2020). However, economic prosperity results in severe ecosystem destruction due to the overexploitation of resources (Wang et al. 2019). These environmental problems in turn also pose a threat to the sustainable development of urbanization and the economy. Therefore, how to balance socioeconomic development with the environment has become a critical issue (Zeng et al. 2016). It is therefore vital to evaluate ecosystem health to support sustainable development and ecosystem management policies. The health of ecosystems has been studied at many different scales and in different habitats, including provinces (Meng et al. 2018), large cities (Su and Fath 2012), urban agglomerations (Kang et al. 2018), rivers (Cheng et al. 2018), wetlands (Chi et al. 2018), and forests (Styers et al. 2010). Currently, there are few studies that examine the association between ecosystem health and its influencing forces, which cannot reflect the degree of interaction and influence between humans and the environment.

The health of a regional ecosystem comprises its ability to maintain its structure and function and to sustainably provide ecosystem services that are influenced by human intervention; these factors represent the most effective method for evaluating the quality of an ecosystem (Constanza 1992; Kang et al. 2018). Three types of analysis frameworks have been proposed thus far for regional ecosystem health assessments: the subsystem, the PSR model (P is pressure, S is state, R is response) (Sun et al. 2016), and the VORS model (V represents vigour, O represents organization, R represents resilience, and S represents ecosystem service) (Spiegel et al. 2001). Early efforts picked the index from the compound subsystem of resource-environment-society-economy (Meng et al. 2018). During the ecosystem health evaluation, we focused on the causal relationship between human activities and ecosystem quality, which resulted in the development of various indicators, including the PSR system and the DPSIR system. While both groups are capable of monitoring ecosystem status and external disturbances, neither group is capable of measuring ecosystem service provision. It is, however, possible to overcome this obstacle by utilizing the VORS functions. VORS tries to define ecosystem health in terms of the quality of both naturalistic ecosystems and ecological services for human beings; it is based on the VOR model. VORS is described using four main components: V is vigour, O is organization, R is resilience, and S is ecosystem services. Vigour is an indication of the metabolism, primary productivity, and activity of a regional ecosystem; organization is an indication of the number of interactions between the different subecosystems; a resilience component describes how well an ecosystem can adapt to external disturbances; and the ecosystem service function component highlights the provision of ecological services influenced by spatial adjacency relationships among different ecosystems (Constanza 1992; Rapport et al. 1998). In this paper, the VORS framework was used to evaluate the ecosystem health of Inner Mongolia in light of the comprehensively measured natural ecological states.

There is very limited research into how environmental factors impact ecosystem health. As a way of addressing this issue, Feng et al. (2016) identified environmental factors and ESs from a global perspective in an attempt to demonstrate their relationship. Qiu and Turner (2013) found that ESs are correlated both on the local (cell) scale and at the landscape scale, and they identified a series of significant explanatory variables. Based on a study of global regression without accounting for geographical variations in regression parameters, their findings represent an average for all of the study areas. Because two closely related factors, precipitation and temperature, were found to have a highly irregular spatial distribution (Turner et al. 2013), the correlations between ecosystem health and these factors show spatial heterogeneity as well (Zhang et al. 2020). Global regressions, for instance, ordinary least squares regression (OLS), when calculated using only the “average” or “global” percentages of parameters, hide information about the local characteristics of the relationship between the variables and thus obscure the real phenomenon (Fotheringham and Brunsdon 2010). Since an increasing number of studies have proven that the geographically weighted regression model can address the aforementioned problem (Han et al. 2019; Zhang et al. 2020), local regression has gradually taken the place of global regression and has become a tool of spatial relationship analysis in ecological processes. Generally, the geographically weighted regression (GWR) model is considered an improvement over conventional linear regression and is used extensively in economics, geography, meteorology, and other fields. This statistical method considers spatial heterogeneity and constructs a local regression equation at each grid to reflect the spatial characteristics of the relationship (Comber et al. 2022). Multiple variables in an ecosystem can be analysed using this method, thus providing more detailed spatial information. Additionally, the GWR model specifies the fitting coefficient of the local model based on the coefficient of each function variable for each geographic location and estimates the parameters of the studied factors. Performing a comparative analysis of the spatial variation of variable coefficient estimates allows the GWR model to produce a more accurate summary of the spatial variation of variable regression coefficients. Hence, the GWR model can solve the problem of geospatial nonstationarity and can better reflect regional differences in influencing factors in geographical and ecological environments. As the GWR method can be applied to geographical data, it has been widely used in landscape ecology. The GWR model, however, still has some shortcomings, especially in regard to exploring the continuity of time variation and the spatial scale of effects. A major disadvantage of GWR is that it only considers the spatial relationship of one piece of time-sequence data, which is not enough to study time-series data. Additionally, the optimal bandwidth found by GWR is the same for each explanatory variable. However, there is no consistency between the effect scales of different explanatory variables when there are multiple explanatory variables. Different explanatory variables do not have the same action scale, which is why it is necessary to find a different optimal bandwidth for each variable for model analysis (Fotheringham et al. 2017; Yang et al. 2019). Previous studies concerning ecosystem health and its driving forces in Inner Mongolia are insufficient. We adopted the VORS model using a 2 × 2 minimum spatial grid to evaluate ecosystem health in Inner Mongolia. In addition, we applied the GWR model to quantify the spatial nonstationary response of influencing factors on ecosystem health. This novel exploration can fill the research gap in the study area.

As part of this article, we examined regional differences in ecosystem health using the VORS model at the 2 × 2 minimum spatial grid scale from 1995 to 2020 and analysed the spatial correlations between ecosystem health and its influencing forces related to meteorology, socioeconomics, and resource endowment using a GWR model at the county level, which is the basis for decision-making related to ecological management in Inner Mongolia. The aim of this study was to determine (1) the spatial heterogeneity of ecosystem health in Inner Mongolia and (2) the nonstationary spatial relationship correlation within ecosystem health and influencing forces investigated. The results of our study will be helpful in forming a conservation policy to conserve the environment at the study site.

## Materials and methods

### Study area

**Fig. 1:**
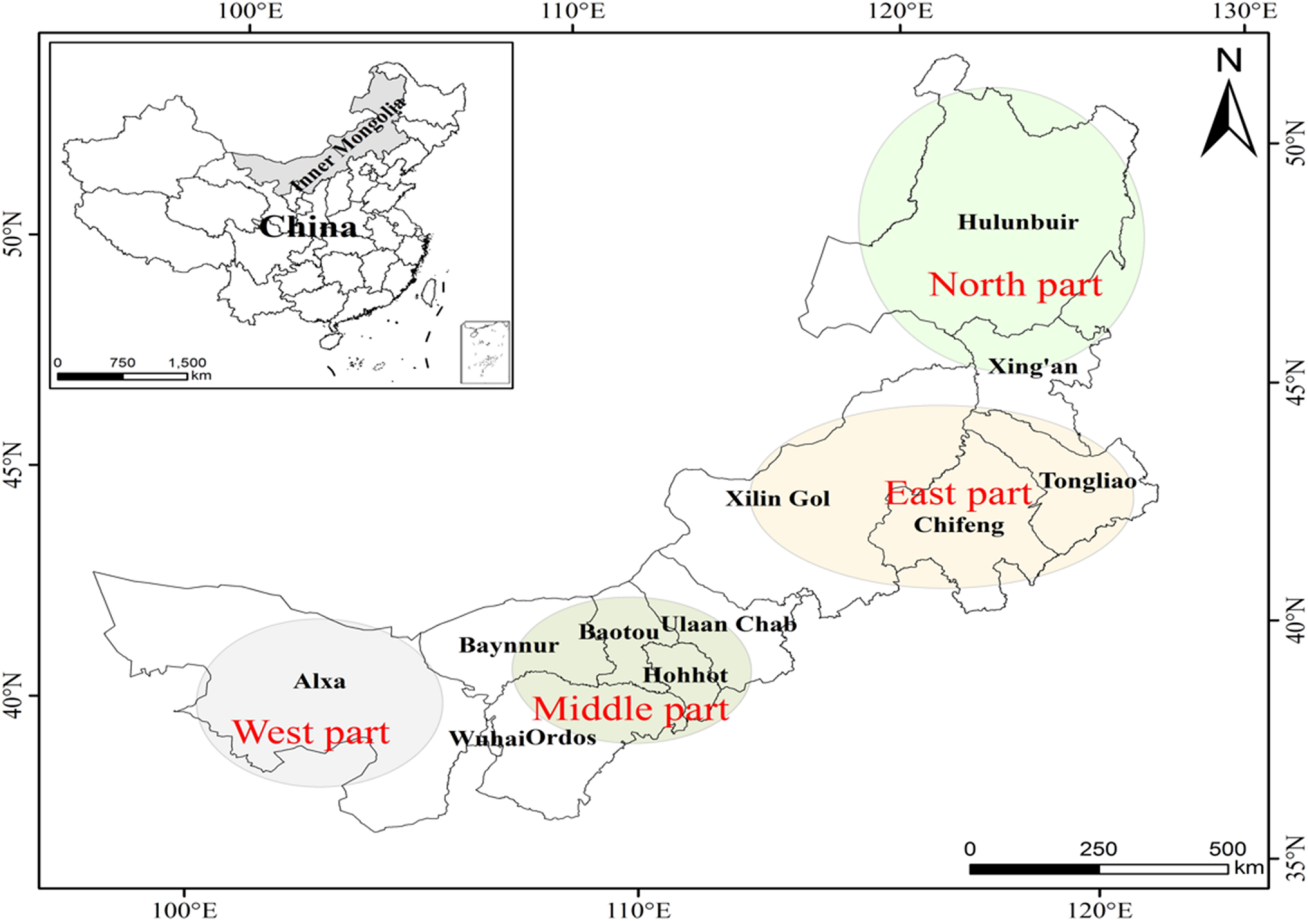
Geographic location and spatial extent of the study area

Inner Mongolia is a typical arid and semiarid region situated in northern China (116°42′E, 43°38′N) characterized by a mid-temperate continental climate, and it has a surface area of approximately 1180.000 km^2^ (Fig. 1). The temperature in the northeastern part of Inner Mongolia fluctuates from − 25.1 to 20 °C, while in western Inner Mongolia, temperatures range from 10 to 20 °C. Rainfall varies from 106.1 to 373.4 mm in an annual cycle, and 82% of the country’s water resources are located in the east, while water resources in the middle and west are not sufficient. Therefore, four distinct regions can be identified within Inner Mongolia in accordance with their climates and land use patterns. The northern part comprises Hulunbuir and Xing’an, the eastern part stretches from Tongliao city to Xilin Gol city, the middle part stretches from Ulaan Chab to Baynnur, and the western part comprises Alxa. Following exploitation of its western region, in the past few decades, Inner Mongolia has experienced rapid economic and urban growth; the growth of coal mining has been largely responsible for this development and has led to a severe degradation of ecosystems. Therefore, it is both urgent and necessary to formulate targeted policies aimed at protecting the environment; the assessment of ecosystem health is essential for making a distinction between the formulation of ecological protection policies and the development of ecological civilization in Inner Mongolia.

### Data sources

Generally, there are two primary categories of data, spatial datasets, and statistical datasets. Spatial datasets were provided by the Resources and Environmental Science Data Centre and National Academy of Sciences of China (available at http://www.resdc.cn) and included LUCC data (with a spatial resolution of 1 km), climate data with a spatial resolution of 1 km × 1 km, average annual precipitation and average annual temperature data, normalized difference vegetation indicator (NDVI), county boundaries of the administrative area in Inner Mongolia, and the digital elevation model (DEM) with a spatial resolution of one kilometre.

The statistical datasets concerning GDP density, population density, and urbanization ratio were gathered from the Inner Mongolia Statistical Yearbook (1995–2020). The data were preprocessed using the ArcGIS10.5 and Fragstats4.2 software. Using the Fragstats4.2 software, we calculated the landscape indices of ecosystems.

### Methods

#### Assessment of ecosystem health

Ecosystem health can be directly measured and fully assessed by its four main aspects: vigour, organization, resilience, and services. By applying the VORS framework developed by Peng et al. (2015), we evaluated ecosystem health at the regional matrix. It should be noted that it is important to normalize each element to a value ranging from 0 to 1. The formula for calculating the ecosystem health index is as follows:

1 $$EHI=\sqrt[4]{EV\times EO\times ER\times ES}$$

In this case, the ecosystem health indicator (EHI) ranks 0–1 depending on how healthy each ecosystem is. The ecosystem health index is divided into five categories using the equal-interval method: highest health (value from 0.8 to 1), suboptimal health (value from 0.6 to 0.8), average health (value from 0.4 to 0.6), unhealthy (value from 0.2 to 0.4), and degraded (value from 0 to 0.2). Ecosystem vigour represents net primary production as well as the metabolism of ecosystems. This paper quantifies ecosystem vigour by utilizing the NDVI (normalized difference vegetation index), which has been extensively utilized in ecosystem health assessments due to its ability to assess the characteristics of eco-environments (Peng et al. 2017; Liao et al. 2018).

Ecosystem organization describes ecosystem complexity as well as structural stability. In this paper, landscape pattern indicators were used to evaluate ecosystem organization and included factors such as connectivity and heterogeneity of the landscape (He et al. 2019). Specifically, our reflection of landscape heterogeneity was represented by the mean patch fractal dimension (MPFD) and Shannon’s diversity index (SHDI). The landscape connectivity index includes two main components: the first is the connectivity of an overall landscape determined by the landscape contagion as well as fragmentation index, and the second is the connectivity of important ecological patches (forests, streams, grasslands) determined by the cohesion and fragmentation index. Additionally, according to previously documented studies and the advice of experts, the weight of overall landscape connectivity is 0.35, the connectivity of ecological patches weight is 0.30 and the landscape heterogeneity weight is 0.35 (Pan et al. 2020).

Specifically, the following is the calculation method:

2 $$\begin{array}{l}EO=0.35LH+0.35LC+0.30IC=(0.25SHDI+0.10MPFD)\\+\left(0.25FN_1+0.10CONT\right)+\left(0.07FN_2+0.03COHE_1+0.07FN_3\right.\\\left.+0.03COHE_2+0.07FN_4+0.03COHE_3\right)\end{array}$$

where *EO* means ecosystem organization. *FN*_*1*_, *FN*_*2*_, *FN*_*3*_, and *FN*_*4*_ represent the landscape fragmentation indicator, forestland fragmentation index, grassland fragmentation index, and water fragmentation index, respectively; *CONT*, *COHE*_*1*_, *COHE*_*2*_, and *COHE*_*3*_ represent the landscape contagion index, forest cohesion index, grassland cohesion index, and water cohesion index, respectively.

Ecosystem resilience refers to the ability of an ecosystem to remain structurally stable regardless of the interference of human beings or any external factors (Rapport et al. 1998). The area-weighted ecosystem resilience coefficient (ERC) is a measure of ecosystem resilience for all kinds of land use. Specifically, based on specialist knowledge and relevant studies (Peng et al. 2017; Pan et al. 2020), the ecological resilience coefficient is determined. The exact calculation formula is as follows:

3 $$ER=\sum_{i=1}^{n}{A}_{i}\times ER{C}_{i}$$

where the resilience of the ecosystem is abbreviated as *ER*, *n* stands for the number of different types of land use, and *A*_*i*_ reflects the area proportion of land use type *i.*

Ecosystem services describe the capacity of ecosystems to generate products and benefits for humankind. An ecosystem service can be analysed and measured in two different ways. The first is by evaluating the coefficients of ecosystem services provided by different land uses (Xie et al. 2017), which is obtained by comparing the ecosystem service value of a given land use type with that of all land uses. Second, spatial neighbouring coefficients differ among land uses, which depends on Inner Mongolia’s actual situation and the related literature. The specific calculation formula is as follows:

4 $$ES=\sum_{j=1}^{n}ES{C}_{j}\times \left(1+\frac{SN{E}_{j}}{100}\right)/n$$

where *ES* refers to ecosystem services, the *ESC *_*j*_ coefficients are the coefficients describing the ecosystem services provided by pixel* j*, and *SNE *_*j*_ can be defined as the total correlation coefficient between two spatial neighbours of pixel *j*. *n* indicates the number of pixels.

#### Spatial correlation test

1. Spatial autocorrelation analysis was applied to investigate the spatial dependencies of ecosystem health and its agglomeration pattern in Inner Mongolia. Spatial autocorrelation consists of both global autocorrelation and local autocorrelation and can therefore indicate the degree to which the attribute of one area is dependent on the attribute of another. To identify the spatial agglomeration of the entire research area, Moran’s *I* index was utilized, as shown in Eq. (5) (Moran 1950). LISA (Anselin 1995) (an indicator of spatial association at a local level) is widely used to measure the spatial association between the value of one attribute and the value of the adjacent attribute (Eq. (6)).
  5 $${{Moran}}^{\mathrm{^{\prime}}}{s}\;{I}=\frac{\sum\limits_{\mathrm{i}=1}^{\mathrm{n}}\sum\limits_{\mathrm{j}=1}^{\mathrm{n}}{\mathrm{W}}_{\mathrm{ij}}\left({\mathrm{x}}_{\mathrm{i}}-\overline{\mathrm{x} }\right)\left({\mathrm{x}}_{\mathrm{j}}-\overline{\mathrm{x} }\right)}{{\mathrm{S}}^{2}\sum\limits_{\mathrm{i}=1}^{\mathrm{n}}\sum\limits_{\mathrm{j}=1}^{\mathrm{n}}{\mathrm{W}}_{\mathrm{ij}}}$$
  6 $$Local\;Mora{n}^{^{\prime}}s\;I=\frac{n\left({x}_{i}-\overline{x }\right)\sum\limits_{j=1}^{m}{w}_{ij}\left({x}_{j}-\overline{x }\right)}{\sum\limits_{i=1}^{n}{\left({x}_{i}-\overline{x }\right)}^{2}}$$

where *n* represents the overall number of grids in Inner Mongolia; *m* represents the number of grids located geographically next to grid *j*; *i* ≠ *j*; $s=1/n{\sum }_{i=1}^{n}{(xi-\overline{x})}^{2}$ *x*_*i*_ and *x*_*j*_ indicate the ecosystem health value of grids *i* and *j*; and *x* stands for the mean value of ecosystem health. The parameter* I* value varies from − 1 to 1, and the absolute magnitude of the* I* index is in accordance with the degree of spatial autocorrelation. When *I* > 0, the correlation between spatial variables is positive; when *I* < 0, the correlation between spatial variables is negative; and when *I* = 0, no spatial relation exists. There are four kinds of local autocorrelations that are considered in this study: high-high (HH), high-low (HL), low–high (LH), and low-low (LL). Units with a high level of ecosystem health are encircled by units with a low ecosystem health level, which indicate the aggregate of units that exhibit a high level of ecosystem health and the aggregate of units that exhibit a low ecosystem health level; units with a low level of ecosystem health are surrounded by units with a high level of ecosystem health, accordingly.

#### Modelling the determinants of ecosystem health

##### Variable selection

In this paper, the following factors were selected as candidate variables for examining influential factors of ecosystem health. Based on previous research, seven factors in the fields of meteorology, socioeconomics, and natural resource endowments were considered (Bebianno et al. 2015; Cheng et al. 2018). Specifically, the average annual temperature (AMT) and average annual precipitation (AMP) were used to define the meteorological conditions. Socioeconomic development was measured by per area gross domestic product (GDP), population density (PD), urbanization rate (UR), and land use intensity (LUI). The biodiversity index (BI) was used to reflect resource endowment.

As there is a high correlation between socioeconomic determinants, an unsuitable choice of variables could lead to collinearity. Therefore, this study used ArcGIS exploratory regression to analyse ecosystem health figures and independent variables from 1995 to 2020 in all possible combinations. Based on each regression model, the corresponding bias-adjusted Akaike information criterion (AICc), adjusted *R*^2^, and maximum variance inflation factor (Max-VIF) were obtained. In fact, the three elements are actually tests for selecting the most appropriate regression model based on statistical principles. The first step in selecting a suitable model involved prescreening and identifying regression models whose maximum variance inflation factor (Max-VIF) was below 7.5. In the second stage, the adjusted *R*^2^ was ranked in descending order; the results are displayed in Table 1. According to Table 1 below, the variable combination models AMT + AMP + LUI + BI ranked first in 2000, 2005, 2010, and 2015 and second in the fittest degree in 2020. Furthermore, its counterpart Max-VIF was comparatively smaller. Thus, AMT + AMP + LUI + BI were chosen as the independent variables in this study.

**Table 1:** Selection of independent variables

	Variable combination	Adjusted *R*^2^	AICc	Max-VIF
1995	AMT + LUI + BI	0.831	− 267.190	2.375
	LUI + BI	0.816	− 259.638	1.035
2000	AMT + AMP + LUI + BI	0.851	− 281.053	1.975
	AMP + LUI + BI	0.842	− 276.541	2.742
2005	AMT + AMP + LUI + BI	0.865	− 273.130	1.430
	AMP + LUI + BI	0.852	− 264.941	2.702
2010	AMT + AMP + LUI + BI	0.838	− 262.537	1.588
	AMP + LUI + BI	0.820	− 253.373	2.769
2015	AMT + AMP + LUI + BI	0.866	− 271.667	2.057
	PD + AMP + AMT + LUI + BI	0.835	− 267.095	2.214
	GDP + AMP + AMT + LUI + BI	0.813	− 245.598	2.501
2020	AMP + UR + LUI + BI	0.893	− 296.267	2.579
	AMT + AMP + LUI + BI	0.886	− 289.108	1.706

##### Geographically weighted regression model

First, the factors of the health ecosystem were examined using ordinary least squares (OLS). In the OLS regression model, dependent and independent variables are assumed to behave the same in all locations within the given geographical area. Hence, the model is unable to measure the nonstationarity of spatial distributions of ecosystem health. In this regard, the estimation of parameters via the OLS model tends to be biased and inefficient. Next, we used a model with variable parameters (GWR) (Fotheringham et al. 2003), which tests whether variables are spatially interconnected across locations.

The OLS regression model is described by Eq. (7):

7 $${y}_{i}={\beta }_{0}+\sum {\beta }_{j}{x}_{i}+{e}_{i}$$

where *yi* represents the ecosystem health value for the *i*th county, and *xi* is its determinant. Similarly, *β*0 represents the constant, *βj* represents the coefficient that should be estimated for the dependent variable, and *ei* represents the stochastic error term. As shown in Eq. (8), the GWR model can be described as a modification of Eq. (7):

8 $${y}_{i}\left({u}_{i},{v}_{i}\right)={\beta }_{0}\left({u}_{i},{v}_{i}\right)+\sum {\beta }_{j}\left({u}_{i},{v}_{i}\right){x}_{i}+{e}_{i}\left({u}_{i},{v}_{i}\right)$$

where (*ui*, *vi*) indicates the geographic location or the geographical site coordinate (i.e., counties). In this study, *ui* and *vi* are the longitudes and latitudes of the *i*th county’s centre point, respectively. GWR is a model that fits datasets of observations near a specific county, resulting in a set of parameters that can be estimated separately for each county. GWR not only estimates parameters for each observation individually but also provides greater observed data (i.e., counties) near the centre, rather than for locations at a greater distance.

Using the GWR model, the estimated coefficients can be written as follows:

9 $$\widehat{\beta }\left({u}_{i},{v}_{i}\right)={\left({X}^{^{\prime}}w\left({u}_{i},{v}_{i}\right)X\right)}^{-1}{X}^{^{\prime}}w\left({u}_{i},{v}_{i}\right)Y$$

where *w*(*ui*, *vi*) is a diagonal spatial weight matrix per observation (i.e., county). A weight matrix for spatial patterns represents the central idea of the model. The parameter value is determined by the bandwidth and geographic location, which is used to describe the nonstationarity characteristics for a location (Poudyal et al. 2012; Yang and Wong 2013). In our case, we utilized ArcGIS 10.5 to assess the GWR and OLS models. In GWR models, Gaussian functions were used to assign weight in space matrices, and across verification processes, the appropriate bandwidth was determined to reduce the Akaike information criteria (AICc).

## Results

### Spatial distribution of EHI

**Fig. 2:**
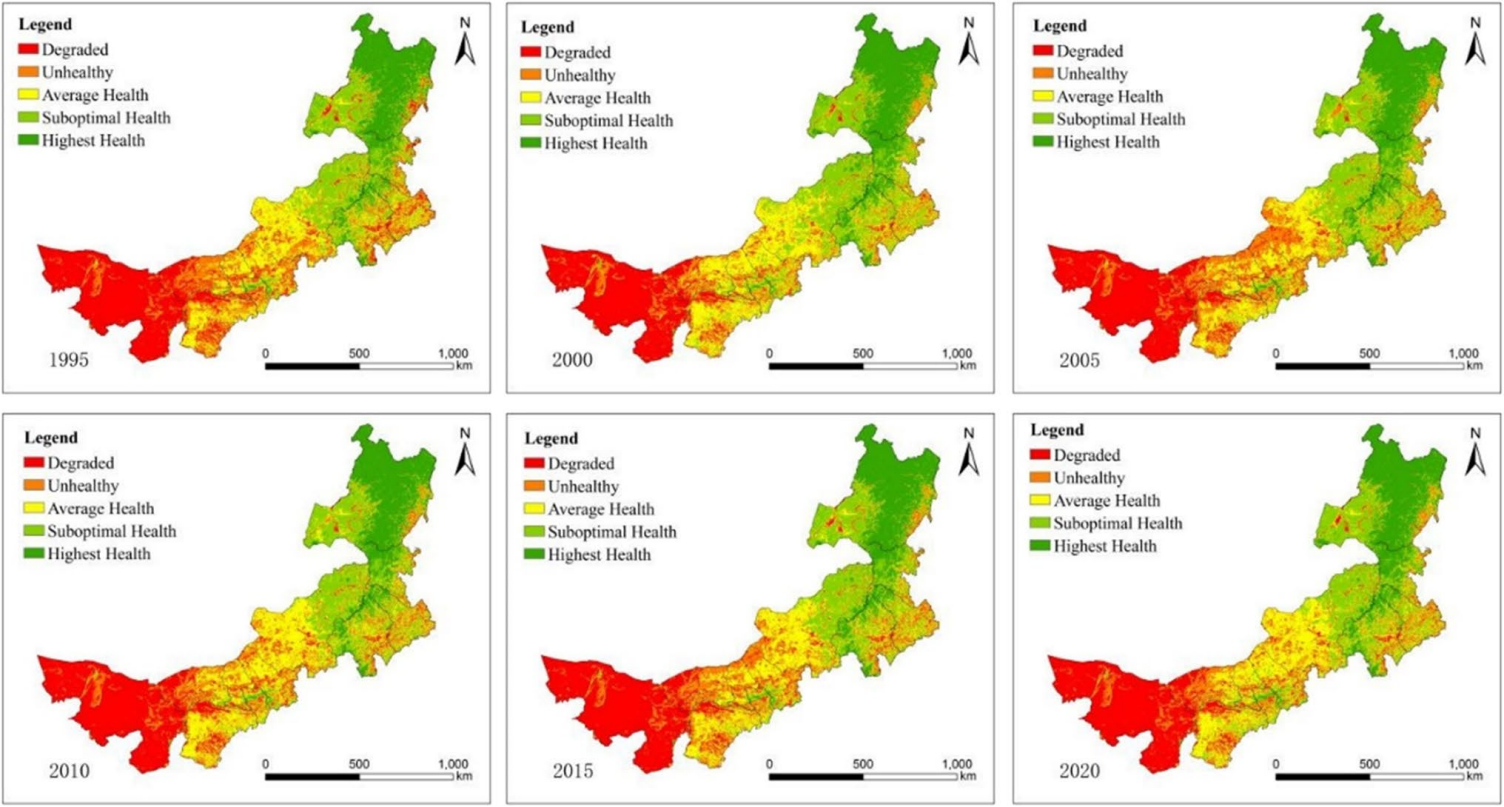
Spatial distribution of ecosystem health in Inner Mongolia from 1995 to 2020

As shown in Fig. 2, ecosystem health in Inner Mongolia increased from west to north from 1995 to 2020. The high-level areas, including the highest health and suboptimal health, were mostly distributed in the northern part (Hulunbuir and Xing’an) and in the eastern part of the region (from Tongliao to Xilin Gol), which was an area with high vegetation coverage and ecological integrity. In contrast, the low-level areas, including unhealthy and degraded areas, were located in the west (Alxa) of the region, where there is no vegetation. However, most of the average level was concentrated in the middle part (from Ulaan Chab to Baynnur) of the region.

The ecosystem health value changed at different rates in different regions. Specifically, there was a downwards trend in western ecosystem health, from 0.26 in 1995 to 0.21 in 2020, and the state of ecosystem health was at degraded levels. Urbanization and economic development in this area have caused extensive expansion of urban construction. As a result of this urban expansion, the regional landscape has been profoundly transformed by excessive population growth, limited land resources, local natural ecosystem alteration, and pollution without effective pollution prevention (Myagmartseren et al. 2017). There are many cities with low incomes where the government is not able to fulfil all of its responsibilities, leading to a shortage of services and facilities (Hardoy et al. 2013). Moreover, in urban areas, vegetation is being replaced by concrete, asphalt, and other hard surfaces that have a much lower potential heat storage, resulting in an increase in land surface temperature (Guan et al. 2016). 2) The ecosystem health mean value in the eastern and middle regions declined from 0.53 in 1995 to 0.52 in 2020, essentially stabilizing at the average level. The mean value of ecosystem health in the eastern and central regions indicated a declining trend, dropping from 0.53 in 1995 to 0.52 in 2020, which was fundamentally maintained at the average health level. Liu et al. (2010) confirmed that the grassland in the middle of Inner Mongolia is degrading, which requires intervention. Climate conditions play a major role in plant growth, especially in arid and semiarid regions such as Inner Mongolia (Li et al. 2006). According to the China Meteorological Administration, in Inner Mongolia, most grass withered in 2014 due to the lack of precipitation at a crucial time in the grass growing period. Chuai et al. reported that during the period from 2000 to 2007, temperature increases and precipitation reductions caused damage to shrubs, pastures, and steppes (Chuai et al. 2013). 3). In 1995, the mean score of ecosystem health in the north was 0.77; by 2020, this value increased to 0.79, primarily remaining at a high level. Over the past few years, in concert with the application of the primary function zone project and the Grain for Green Project, there has been a gradual decrease in the amount of interference imposed by humans on the natural ecosystem of these areas (Yang et al. 2020). Therefore, these areas have improved their ecological carrying capacity and the restoration of vegetation.

### Spatial autocorrelation of EHI

The global Moran’s indicator values were 0.6638, 0.6615, 0.6607, 0.6573, 0.6568, and 0.6432 in Inner Mongolia from 1995 to 2020, respectively, which were significant at the 1% level. It is evident that ecosystem health exhibits significant spatial autocorrelation in Inner Mongolia. Grids with equivalent ecosystem health showed significant spatial agglomeration effects, and the agglomeration degree steadily declined from 1995 to 2020. The results show that the ecosystem health index exhibits a significant spatial agglomeration effect in Inner Mongolia, which means that neighbouring areas have an impact on regional ecosystem health; areas with high ecosystem health indices are adjacent to each other, while areas with low ecosystem health indices are also adjacent to one another. According to the data, Moran’s *I* experienced a significant decline in 2020, suggesting that there is a decline in the spatial agglomeration degree of Inner Mongolia’s ecosystem health, which indicates that the regional ecosystem health is gradually moving towards a state of balance in the region. This state can be attributed to the fact that all counties and cities throughout the region have intensified their efforts to improve ecological protection during the “Twelfth Five-Year Plan,” as well as the fact that areas with low ecosystem health have also escalated their implementation of various policies and measures, contributing to the creation of a high-quality ecological environment with low ecological pressure, good ecological conditions, good air quality, and green waters and mountains.

**Fig. 3:**
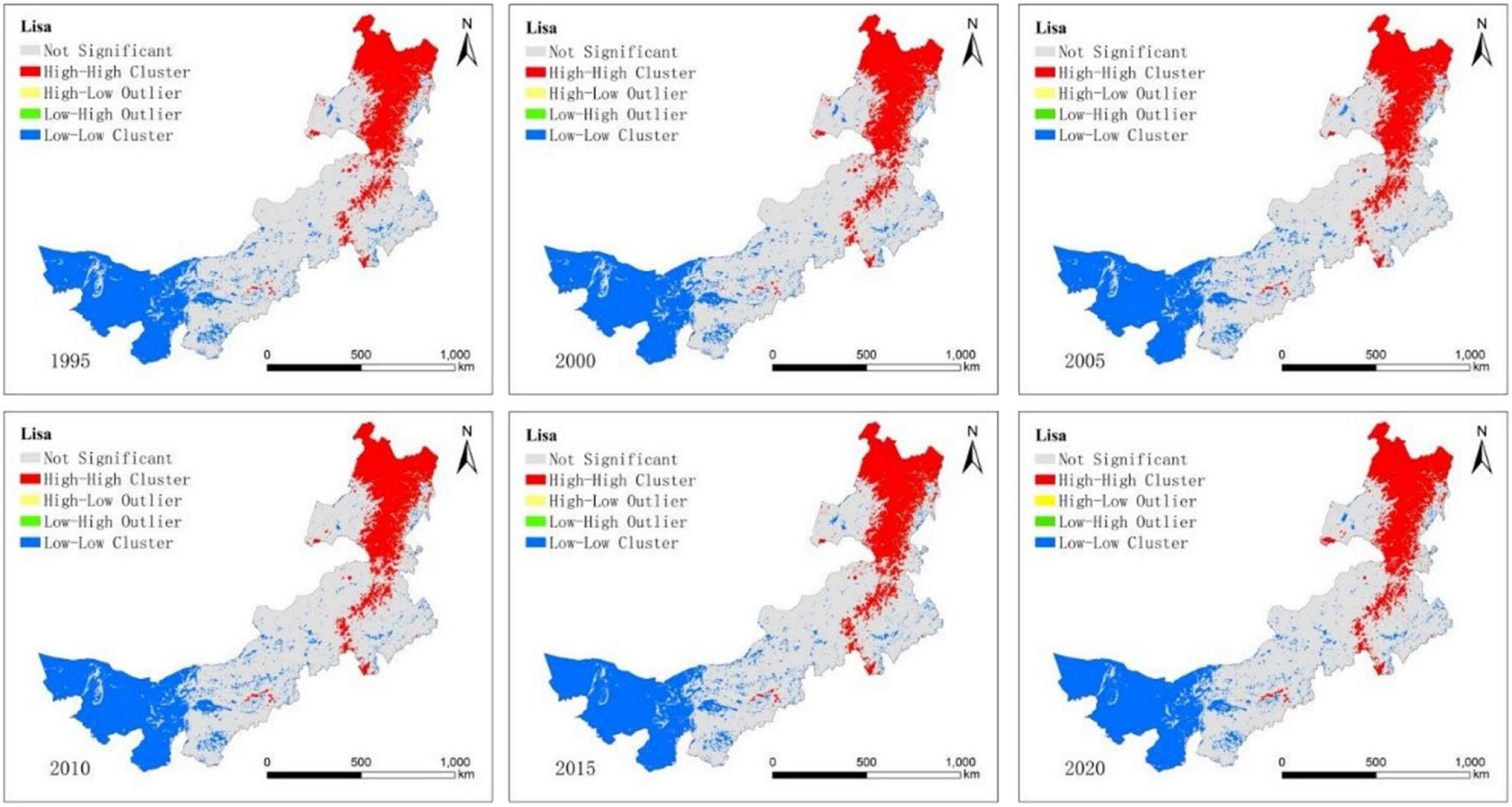
The cluster map of spatial association for EHI in Inner Mongolia from 1995 to 2020

This map illustrates that two parts of the region showed positive spatial autocorrelation (high-high or low-low) at the 5% significance level. Negative spatial autocorrelation (low–high or high-low) was not observed in the research region from 1995 to 2020. The high-high type was mainly situated in the northeastern region, while the low-low type was distributed in the western region and coal mining area. During the research period, the LISA clustering pattern of mean ecosystem health barely changed (Fig. 3).

### Factors influencing the EHI

#### Model diagnosis

To better compare the performance of the GWR and OLS model’s predictive ability and the ability to solve spatial autocorrelation problems, indicators such as adjusted *R*^2^, AIC value, and spatial autocorrelation of residual were used to compare and analyse the GWR and OLS results, as shown in Table 2. Model predictive power is commonly described by the AIC and adjusted *R*^2^. Generally, the higher the adjusted *R*^2^ value is, the better the variance is explained, and the lower the AIC value is, the closer the situation is to reality. The spatial autocorrelation of residuals from the GWR and OLS models can be quantified by Moran’s *I* index. The assumption of using OLS is then violated if the model residuals show significant spatial autocorrelation, and its validity is questioned. Due to the spatial heterogeneity of the driving mechanism, the OLS model did not fit well for adjusted *R*^2^. Based on the adjusted *R*^2^ of the GWR model, both values were higher than the corresponding OLS model, suggesting that the GWR model is better able to explain the impact of the influencing factor on ecosystem health. As shown in Table 2, compared to the OLS model, the GWR model has a lower AIC, which indicates that it is more effective at quantifying the correlation between ecosystem health and influencing factors. In light of these findings, it can be concluded that GWR has greater explanatory ability than the OLS model. The Moran’s *I* index of the OLS model varied between 0.339 and 0.417, suggesting that there may be a positive spatial correlation between the variables. Compared with the OLS model, the Moran’s *I* index of the GWR model is smaller than that of the corresponding OLS model, which indicates that the GWR model not only takes into account the spatial autocorrelation of variables but also shows that GWR can better solve the problem of spatial autocorrelation. As a result, the GWR model was found to be robust and outperformed global regression when explaining correlations between ecosystem health and influencing factors in our study.

**Table 2:** Statistical test comparison of OLS and GWR from 1995 to 2020

	AICc		Ajusted *R*^2^		Moran’s *I*	
	AIC_o_	AIC_G_	*R*^**2**^_o_	*R*^**2**^_G_	Moran_o_	Moran_G_
1995	− 267.342	− 290.769	0.833	0.869	0.417	0.386
2000	− 281.053	− 316.044	0.851	0.895	0.339	0.224
2005	− 273.130	− 302.850	0.865	0.900	0.374	0.324
2010	− 262.537	− 290.834	0.838	0.878	0.417	0.346
2015	− 271.667	− 306.255	0.866	0.906	0.356	0.351
2020	− 282.364	− 319.204	0.892	0.912	0.396	0.367

AIC_O_ and AIC_G_ are the Akaike information criterion for OLS and GWR; *R*^2^_O_ and *R*^2^_G_ are the coefficient of determination value for OLS and GWR; Moran_o_ and Moran_G_ are the Moran’s *I* value for OLS and GWR.

In addition, there are interregional spatial correlations and spatial heterogeneities due to the uneven distribution of natural resources and socioeconomic factors. As a result, the assumptions related to the global regression model are no longer valid, such as the assumptions that data values are independent of geographical location, no spatial correlation exists, and sample data is equally distributed. In reality, the global regression model estimates regression parameters based on the average value of the entire region, which has no local significance and cannot reflect the true characteristics of the regression parameters. Thus, using global overall parameters cannot properly account for individual cases and, in this case, spatial heterogeneity. To address these issues, a GWR was applied in this study to estimate local regression on adjacent subsamples within each group.

#### Estimates produced by the GWR model

**Fig. 4:**
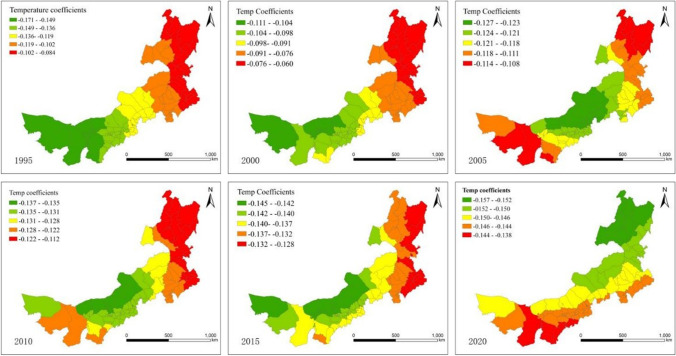
Spatial variability of the geographic regression coefficients of Temp from 1995 to 2020

**Fig. 5:**
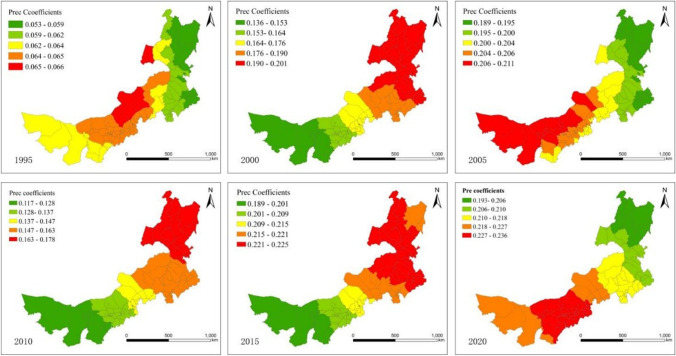
Spatial variability of the geographic regression coefficients of the PREC from 1995 to 2020

**Fig. 6:**
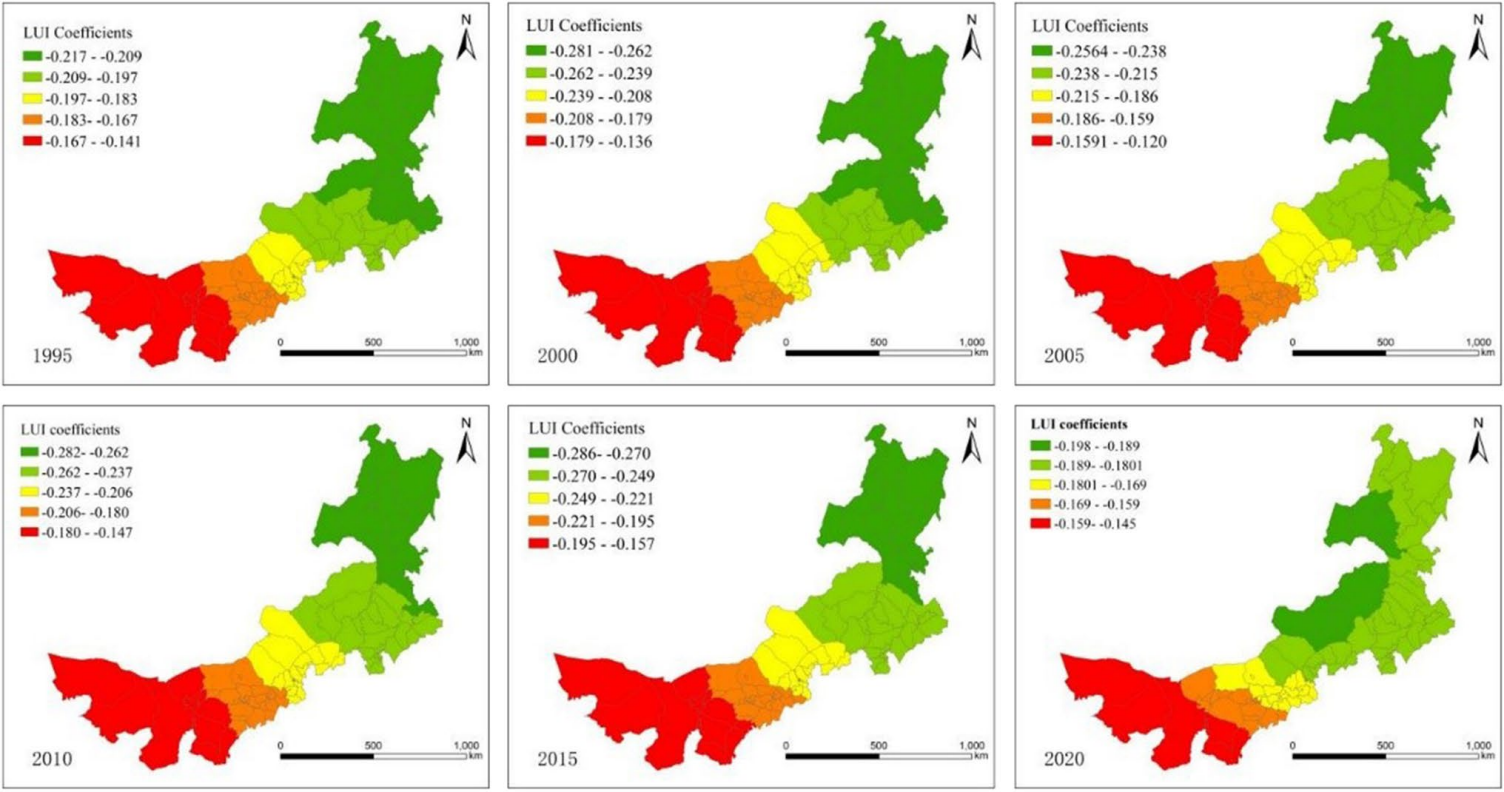
Spatial variability of the geographic regression coefficients of the LUI from 1995 to 2020

Based on the GWR model outcomes, we can conclude that the regression coefficient values varied between different regions. Corresponding local coefficients for Temp, PRE, LUI, and BI varied between counties on the EHI, illustrating the obvious spatial heterogeneity between the EHI and the influencing factors. Based on the regression coefficients of each factor, the nonstationary spatial distributions of ecosystem health to the influencing factors were plotted (Figs. 3–6). Positive regression coefficients indicate that increased influencing factors will result in an increased likelihood of ecosystem health, while negative regression coefficients indicate that an increase in the influencing factors is likely to lead to a decline in ecosystem health.

Figures 4 and 5 illustrate the spatial relationship between the EHI and meteorology, including annual mean temperatures and annual mean precipitation. According to the corresponding regression coefficient maps, ecosystem health is adversely affected by temperature; in other words, temperature exerts a more pronounced negative mechanism of action on regional ecosystem health in overall Inner Mongolia region, meanwhile, the spatial distribution has significant band-ring spatial characteristic. Spatially, the overall distribution of the absolute values of the annual mean temperature regression coefficients showed a gradual increase in the northeast to the southwest directions, indicating that the influence of precipitation on the health of ecological environment in southwest is greater than that in northeast region. Despite the significantly negative impact of the region being relatively unchanged, there are differences in component counties between different time periods. Among the northeastern counties with low temperature conditions, the EHI showed the least sensitivity to temperature, demonstrating the importance of vegetation in regulating temperature. Middle or western parts of the region with higher temperatures and less rainfall had a more significance degree of negative impact, indicating ecosystem health in this region is more sensitive to temperature. Precipitation has a significant positive effect on the variation of ecosystem health in the study area with significant block-like spatial distribution characteristics. In terms of regression coefficients, the coefficient of the precipitation factor is positive throughout the region. From the perspective of the spatial distribution trend of driving intensity, influence pattern shows a gradual increase from southwest to northeast in 2000, 2010, and 2015, and the overall block characteristics are significant, indicating that the more precipitation, the higher the ecosystem health. The most prominent and positive responses of regional ecosystem health to precipitation were observed in the northeastern states of Inner Mongolia, whereas the lowest responses were observed in the western counties with higher temperatures and lower precipitation. In general, the model results concluded that the climate in Inner Mongolia was the most significant determinant affecting ecosystem health. Our findings are in agreement with the conclusions of previous research in China (He et al. 2019). The meadow steppe ecosystems in northeastern regions are extremely susceptible to climate fluctuations.

Figure 6 presents the spatial relationship between the LUI and EHI. In Inner Mongolia, the regression coefficient maps indicate that LUI negatively affected ecosystem health from 1995 to 2020. The higher intensity of land use indicates the greater disturbance and input of human activities to the land, which tends to contribute to the formation of production and living space and thus affects the quality of ecological environment. In addition, unreasonable land development tends to lead to land degradation, which in turn affects the health of the ecological environment. In terms of spatial distribution, the absolute values of regression coefficients show that trends of gradually becoming larger from east to west, east, and northeast Inner Mongolia are significantly higher than west and central Inner Mongolia, with significant spatial heterogeneity. There are no positive correlations between LUI and ecosystem health in the study region. In general, the cities with the largest negative impacts are located in the western part of the region, including Alxa and parts of Baynnur and Ordos. The Alxa Plateau refers to an area of severely eroded ancient highlands. There are three deserts in this region: Badain Jaran, Tengger, and Ulanbuh. The main landscapes are deserted mountains, sand-covered deserts, and gobis, which occupy over 70% of the whole area. A primary cause of desertification in Inner Mongolia is land reclamation because of plant composition and wind-exposed soil. These two aspects have resulted in soil nutrient depletion and deterioration of soil structures due to wet weather and water erosion. Hence, fertile soil has become barren since fertile land has been transformed into barren land. Ordos, with the development of urbanization, has become one of the most rapidly growing cities. Economic development was characterized by natural resource extraction and infrastructure construction, resulting in deforestation and ecosystem health disruption. These results implied that land use intensity aggravates the degradation of ecosystem health in the western part of the region. The vegetation coverage in northeast Inner Mongolia is mainly dominated by natural forests, which is extremely sensitive to land use intensity and anthropogenic disturbance, indicating that if ecosystem in this region is threatened with disturbance, it will be difficult to recover and may suffer losses. In general, when the land use intensity is high, increased recreational activities, industrial construction, economic development, and other human-related activities will have a certain negative impact on the overall ecosystem health, and the following high mobility of the urban population will also be destructive to the natural system. In contrast, however, the disorderly development of natural systems at low land use intensity is not necessarily positive. Appropriate human intervention to guide scientific and reasonable planning, shape the landscape, and organize the ecological pattern is also of positive significance to the natural system.

**Fig. 7:**
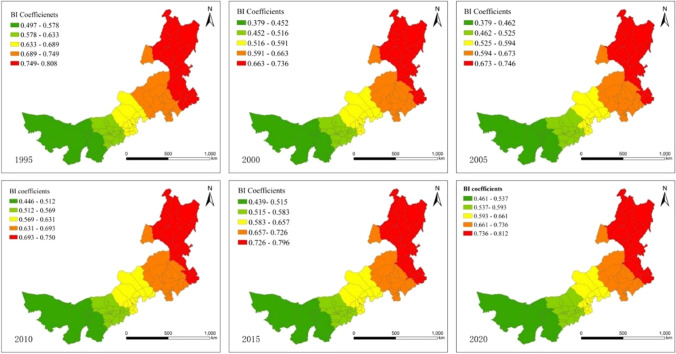
Spatial variability of the geographic regression coefficients of the BI from 1995 to 2020

Figure 7 illustrates the spatial relationship between BI and EHI. The biodiversity index (BI) in Inner Mongolia shows significant positive driving characteristics and spatial distribution trends of patchiness on regional ecosystem health. The spatial distribution of the overall driving characteristics shows that the overall ecosystem health in Inner Mongolia is positively driven by the biodiversity index. The northern and eastern parts of Inner Mongolia are typical significant areas, and the intensity of the positive effect of biodiversity index in the forested land core area is significantly higher than that in the grassland and bare land dominated areas. During the study period, the spatial distributions of the regression coefficients of the biodiversity index were basically the same, but the coefficients increased to varying degrees, which indicated that the positive impact of the BI on the EHI showed an increasing trend, especially after the Grain for Green Project in 2000. This result implies that government policy is beneficial for increasing vegetation coverage and greatly improving the ecological health of the overall region. Specifically, the regression coefficient graphs demonstrate that the counties with a greater positive effect are primarily centralized in the northeastern region, including the provinces of Hulunbuir, Xing’an, and Tongliao. The main driving cause of this phenomenon is the forest ecosystem in these regions as a genetic reservoir of biodiversity. The increase in species diversity and community complexity will directly lead to an increase in the ecosystem health. Therefore, the enhancement and optimization of forest ecosystem in northeast region has a significant role in the overall ecosystem health improvement of Inner Mongolia. The significance degree of the positive impact increases gradually from the west to the north of Inner Mongolia. Previous studies have found that total annual precipitation, which is the primary influence on the NDVI in Inner Mongolia, has a positive correlation with the NDVI. Precipitation is highest in Hulunbuir and the Xing’an League in northeastern Inner Mongolia, and these areas have the highest vegetation cover in Inner Mongolia, where forests and typical steppe are present. Desert is the dominant landscape pattern in the western part of the region, while grassland is mainly found in the middle part of the region. Implementing the Grain for Green project at the start of the twenty-first century has led to the conversion of sporadic farmland into grassland or forests, which is also beneficial for regional ecosystem health.

## Discussion

### Comparison with previous studies

We evaluated the spatial distribution of ESH from 1995 to 2020. The results showed that the areas with relatively weak ESH were mainly located in western Inner Mongolia, which mainly comprises desert areas and rapidly growing socioeconomic areas, indicating that policy-makers should take some strategies to combat desertification, implement industrial transformation, and upgrade energy-intensive industries. Areas with strong ESH were located in northern areas, a region in which many mountains and much forestland are distributed, such as Daxinganling virgin forest, which also plays a certain role in ESH protection. The areas with average ESH were distributed in the eastern and middle parts of the region, suggesting that the classic ecology concepts or theories and the applicable ecological principles of grassland conservation or management should be taken into account in grassland-dominated areas.

In terms of driving forces, the findings of our study show that the ecosystem health level is strongly influenced by both meteorological and socioeconomic factors. This result is in accordance with prior studies that showed that regional ecological sensitivity is heavily determined by climatic factors (Meng et al. 2016; Zhang et al. 2017), and wetland, grassland, and woodland ecosystem changes are affected by the complicated combination of climate and human force (Wan et al. 2018; Li et al. 2019). However, the nonstationary spatial interaction between ecosystem health and the influencing forces has not been sufficient. Until recently, studies of the factors that influence ecosystem health have neglected the point of view that ecosystem constituents are affected by energy transfer and related to information exchange, which displays a notable spatial diffusion impact. Furthermore, urbanization transmits factors of production, including capital, labour, and industry, between areas. That is, the urbanization of adjacent regions adversely affects local ecosystems (Xie et al. 2021). At present, researchers have begun developing an aware of this fact and have begun exploring the spatial variation relationship using the OLS model. For example, Li et al. (2021) explored urbanization’s impact on ecosystem health by applying the GWR model. The effects of land use change on ecosystem services have been examined by Chen et al. (2019) utilizing the GWR model. The GWR model can provide location guidance for decision-making in contrast with the global regression model. The spatial link between the individual locations can be reflected by the local regression equation. Furthermore, previous studies have only paid attention to one specific element concerning urbanization, disregarding other meteorological and socioeconomic elements, which does not permit objectively evaluating the influencing factors on ecosystem health.

### Implications of spatial nonstationary responses

Our study found that ecosystem health in Inner Mongolia was strongly related to climate factors (PRE, TEM), resource endowment factors (BI), and socioeconomic factors (LUI) and that the characteristics and intensity of those correlations demonstrated obvious spatial heterogeneity. Hence, decision-makers may intend to design interventions that can improve synergistic effects but have contrary results in other locations. Our results can help decision-makers identify the spatial distributions of each response, which can be controlled to promote ecosystem health. For example, in this study, we found that a strong correlation exists between temperature and ecosystem health, demonstrating a negative correlation in the study area, suggesting that managers and policy-makers should attempt to decrease the temperature to improve ecosystem health. In a previous study, clustered vegetation was found to be an effective means of regulating the surface temperature (Estoque et al. 2017). Designers should strive for a higher density of forest canopy to minimize evaporation and solar radiation, as precipitation has a positive correlation with ecosystem health in the overall study area. The implementation of precautions, such as monitoring meteorological disasters, should also be conducted to minimize the impact of climate extremes on the ecosystem.

The results of our study indicate that the effect of the BI factor on the EHI is positive in the study area, suggesting that decision-makers should strive to improve ecosystem health through ecological conservation policies. Projects such as Grain for Green and national parks and nature reserves contribute to protecting the environment and managing natural resources. For instance, the Xilin Gol Biosphere Reserve, West Ordos Nature Reserve, Da Qing Shan National Nature Reserve in Northern Hohhot, National Nature Reserve in Heilihe in the boundary area of rural Chi Feng, and the Cheng De Natural Barrier to Protect China’s northern region have a positive influence on natural ecosystems since their grasslands have been maintained and their forests have been cultivated in a positive way. Additionally, the Shelter Belt Construction Project in the Three-North area has assisted with protecting the environment and conserving nature (Rajagopalan et al. 2014). In terms of LUI, the spatial associations between ecosystem health and LUI showed that the proportions of land use categories were definitely associated with ecosystem health deficits and surpluses. The EHI was generally stronger in counties with a greater percentage of pastures and forests. Additionally, urbanization areas and agricultural reclamation displaced the green space, which then resulted in a decline in the EHI or even a deficit. It is therefore vital to optimize land use structure and avoid excessive reclamation and construction. The government should not only make full use of the ecological resources of Inner Mongolia but meantime protect the original landscape ecology. Construction projects must be approved before proceeding, and commercial and industrial land use is strictly limited. This is to protect the ecological environment from damage as much as possible while promoting regional economic development, thereby reducing the significant pressure on ecosystem health caused by excessive land use intensity.

We can adopt different management strategies according to the local conditions in different regions. In the ecologically fragile area of the western desert, combined with the current situation in the region, in the development of mineral resources and the development of energy and chemical industries, the industry access mechanism should be improved to ensure that resource development and ecological restoration are carried out simultaneously. In the central and east regions, realizing the full development of green space; overall planning and rational layout should be made to increase the compactness of industrial land and improve land use efficiency. In the northeastern forest ecological balance zone, the general principle should be conservation-oriented, supplemented by development, using biological resources, developing green and efficient non-polluting industries, and enhancing the economic benefits within the ecological capacity. Policymakers and decision makers should pay more attention to the balance between socioeconomic development and ecological conservation in such semi-arid areas. In addition, ecological background conditions for spatial differentiation should be considered in land use planning.

### Limitations

It is important to note that our study has some limitations. First, although using a local regression model will enable us to estimate the spatial interaction between ecosystem health and influencing forces, the GWR model also has an important limitation, which presumes that the interaction between independent variables and dependent variables differs at the same spatial level. Therefore, it is necessary to investigate interactions on multiple scales among response and explanatory variables. Second, we determined nonstationary relationships between ecosystem health and motivating forces at the administrative units. Despite its convenience for the establishment and application of environmental conservation policies, the relationship between responses may vary at different scales (Su et al. 2020). Thus, we have not analysed these relationships at other scales, as statistical data were not available at the scale of grid cells. If the statistical data were transformed from the level of the administrative division to the level of the grid cell, it would be possible to study the scale effect correlation between the variable and its response. However, in this study, the scale is limited to the district-county scale. Subsequent studies can continue to enhance the data type and precision, thus enabling continued subdivision within district and county units and analysing the mechanism of each influencing factor more accurately and improve the practical significance of the evaluation results.

## Conclusion

In this paper, we evaluated the ecosystem health conditions from 1995 to 2020 and explored the factors contributing to spatial ecosystem health differences. Traditional econometric and spatial models only account for the significance of a specific determinant from a global viewpoint while ignoring the geographic heterogeneity of those indications. In this study, using exploratory spatial data analysis, we examined the characteristics of agglomerations in Inner Mongolia ecosystem health from 1995 to 2020 and utilized the GWR model to assess the different effects of influencing determinants on ecosystem health in the study area.

We obtained the following conclusions from the results. Inner Mongolia’s ecosystem health level improved from northwest to southeast, and there was an upwards trend, followed by a downwards trend from 1995 to 2020. Grids with similar ecosystem health exhibited effects of spatial agglomeration, and the spatial agglomeration degree decreased gradually from 1995 to 2020. Using geographic variation analysis, it was revealed that influencing factors affecting ecosystem health exhibited intensive spatial heterogeneity. In particular, the meteorological factors concerning average annual temperature and average annual precipitation contributed significantly to determining the ecosystem health in each region. Moreover, annual average temperature (AMT) and land use intensity (LUI) were negatively correlated with ecosystem health, whereas the coefficients of annual average precipitation (AMP) and biodiversity (BI) were estimated to be positive, indicating a positive impact on ecosystem health. The counties where ecosystem health can be significantly improved by annual average precipitation (AMP) are predominantly located in the eastern and northern regions, whereas the counties where ecosystem health can be significantly alleviated by annual average temperature (AMT) are predominantly located in the same region. Moreover, LUI exerts a negative influence on ecosystem health in western regions of counties (such as Alxa, Ordos, and Baynnur). The distribution of counties strongly influenced by the biodiversity index gradually increased from the southwest to northeast of Inner Mongolia. By evaluating the relationship between ecosystem health and its influencing force in Inner Mongolia, we can offer guidelines for the functional implementation and management of ecosystems as well as their restoration in different regions.

## Data Availability

The data that support the findings of this study are available on request from the corresponding author upon reasonable request.
